# Bi-specific TCR-anti CD3 redirected T-cell targeting of NY-ESO-1- and LAGE-1-positive tumors

**DOI:** 10.1007/s00262-012-1384-4

**Published:** 2012-12-22

**Authors:** Emmet McCormack, Katherine J. Adams, Namir J. Hassan, Akhil Kotian, Nikolai M. Lissin, Malkit Sami, Maja Mujić, Tereza Osdal, Bjørn Tore Gjertsen, Deborah Baker, Alex S. Powlesland, Milos Aleksic, Annelise Vuidepot, Olivier Morteau, Deborah H. Sutton, Carl H. June, Michael Kalos, Rebecca Ashfield, Bent K. Jakobsen

**Affiliations:** 1 Haematology Section, Institute of Medicine, University of Bergen, Bergen, Norway; 2 Haematology Section, Department of Internal Medicine, Haukeland University Hospital, Bergen, Norway; 3 KinN Therapeutics AS, Haukeland University Hospital, 9th Floor Laboratory Building, Bergen, Norway; 4 Immunocore Ltd, 57C Milton Park, Abingdon, Oxfordshire, OX14 4RX UK; 5 Department of Pathology and Laboratory Medicine, Perelman School of Medicine, University of Pennsylvania, Philadelphia, PA USA; 6 Abramson Family Cancer Research Institute, Perelman School of Medicine, University of Pennsylvania, Philadelphia, PA USA

**Keywords:** Bi-specific TCR, ImmTAC, Cancer immunotherapy, NY-ESO-1, LAGE-1, Time-domain

## Abstract

NY-ESO-1 and LAGE-1 are cancer testis antigens with an ideal profile for tumor immunotherapy, combining up-regulation in many cancer types with highly restricted expression in normal tissues and sharing a common HLA-A*0201 epitope, 157–165. Here, we present data to describe the specificity and anti-tumor activity of a bifunctional ImmTAC, comprising a soluble, high-affinity T-cell receptor (TCR) specific for NY-ESO-1_157–165_ fused to an anti-CD3 scFv. This reagent, ImmTAC-NYE, is shown to kill HLA-A2, antigen-positive tumor cell lines, and freshly isolated HLA-A2- and LAGE-1-positive NSCLC cells. Employing time-domain optical imaging, we demonstrate in vivo targeting of fluorescently labelled high-affinity NYESO-specific TCRs to HLA-A2-, NY-ESO-1_157–165_-positive tumors in xenografted mice. In vivo ImmTAC-NYE efficacy was tested in a tumor model in which human lymphocytes were stably co-engrafted into NSG mice harboring tumor xenografts; efficacy was observed in both tumor prevention and established tumor models using a GFP fluorescence readout. Quantitative RT-PCR was used to analyze the expression of both NY-ESO-1 and LAGE-1 antigens in 15 normal tissues, 5 cancer cell lines, 10 NSCLC, and 10 ovarian cancer samples. Overall, LAGE-1 RNA was expressed at a greater frequency and at higher levels than NY-ESO-1 in the tumor samples. These data support the clinical utility of ImmTAC-NYE as an immunotherapeutic agent for a variety of cancers.

**Electronic supplementary material:**

The online version of this article (doi:10.1007/s00262-012-1384-4) contains supplementary material, which is available to authorized users.

## Introduction

Understanding of anti-tumor immunity has increased significantly over the past decade, including fundamental insights into the role of innate and adaptive immune responses in targeting and eliminating tumors [1, 2]. In both animal studies and, more recently, clinical studies, CD8+ cytotoxic T cells (CTLs) have been shown to be major effector cells involved in the eradication of tumor cells [3–5]. A requisite step for the targeting of tumors by CTL is the binding of T-cell receptors (TCRs) to peptides derived from the proteome of tumors and presented on the cell surface in the context of MHC Class I molecules (pMHCs) [6]. A major limitation for the effective targeting of established tumors by T cells results from central tolerance, whereby T cells with strong affinity for self-antigens are deleted in the thymus [7]. In addition, the natural selection process inherent in the growth and establishment of tumors in immune-competent hosts leads to the selection of tumors with decreased antigen and MHC expression and alterations in antigen processing and presentation, reducing the surface density of pMHC complexes [8]. Finally, peripheral tolerance mediated through host and tumor-driven immunosuppressive mechanisms contributes to the ultimate inability to mount an effective T-cell immune response to target tumors [9–11].

One approach to overcome the lack of potent anti-tumor T-cell immunity is the ex vivo genetic modification of T cells to target tumors through the use of affinity-enhanced receptors generated from either T cell or antibody-derived receptors; recent clinical data have provided evidence that such approaches hold significant clinical promise [12, 13]. A complementary approach that does not require ex vivo manipulation of T cells involves the use of fusion proteins that combine tumor recognition and T cell engaging domains to redirect T cells to target tumors [14]. Among this class of reagents are several monoclonal antibody-anti CD3 scFv fusion reagents, some of which have undergone clinical evaluation with strong evidence of efficacy [14–17]. The use of antibody-based T-cell targeting agents is restricted to targeting of antigens expressed on the cell surface. To overcome this limitation, we have recently developed a class of bi-specific fusion protein reagents termed ImmTACs (Immune-mobilizing monoclonal TCRs against Cancer), which target proteosomally processed epitopes derived from intracellular, surface bound, and secreted antigens [18]. ImmTACs are comprised of soluble, high-affinity (pico-Molar) monoclonal TCRs (mTCRs) specific for antigen-derived pMHC complexes, combined with a humanized anti-CD3 scFv domain, which activates a potent anti-tumor T-cell response. The first clinical candidate ImmTAC product, IMCgp100, specific for gp100_280–288_ is currently undergoing Phase I clinical testing in melanoma patients (trial number NCT01211262).

NY-ESO-1 (CT6, also known as LAGE-2) and LAGE-1 are among the prototypic cancer testis antigens [19, 20]. Although little is known about their functions, normal tissue expression of both of these antigens is principally limited to immune privileged sites [20–22]. Like other CT antigens, both NY-ESO-1 and LAGE-1 are expressed during development as well as by a wide range of tumors including myeloma [23, 24] and a variety of solid tumors such as ovarian [25], non-small-cell lung cancer (NSCLC) [26, 27], and melanoma [28, 29]. NY-ESO-1 has been targeted extensively in clinical trials using a variety of approaches including vaccines and gene-modified T cells [13, 30]. Multiple studies have demonstrated that NY-ESO-1 is antigenic in human patients [21, 31], although vaccination approaches alone rarely lead to a clinically significant response [32, 33].

We have previously described both a high-affinity mTCR and ImmTAC reagent (ImmTAC-NYE) specific for the immunogenic HLA-A2 presented peptide NY-ESO-1_157–165_ (SLLMWITQC) [18, 34]. Notably, since the same epitope is processed from the LAGE-1 antigen [20, 35], ImmTAC-NYE can be used to redirect T cells against HLA-A*0201-positive tumors that express either NY-ESO-1 or LAGE-1 antigens. In this report, we demonstrate that ImmTAC-NYE redirects potent antigen-restricted T-cell activity against NY-ESO-1- and/or LAGE-1-positive tumors, including established cell lines shown previously to present low levels of cell surface epitope [34] and primary lung cancer cells. We show in vivo targeting of high-affinity NYE TCR to tumors presenting the NY-ESO epitope via time-domain optical imaging of fluorescently labelled mTCR. Importantly, we demonstrate ImmTAC-NYE-mediated inhibition of tumor growth in vivo, in a model co-xenografted with both human peripheral blood lymphocytes and cancer cells. The results provide rationale to support the further preclinical and clinical development of ImmTAC-NYE as a bi-specific immunotherapeutic agent to treat HLA-A*0201-positive and NY-ESO-1- and/or LAGE-1-expressing solid cancers.

## Materials and methods

### Human tissues, cells, and cell lines

Primary tumor tissue from lung and ovarian cancer patients was collected at the University of Pennsylvania under an IRB approved protocol. Pathologic review of biopsies confirmed diagnosis in each case. Tumor biopsies were processed as described [36] to obtain single-cell suspensions which were viably frozen.

Cell lines J82 (urothelial), SK-Mel-37, Mel526, Mel624, A375 (melanoma), IM9 (EBV B), and U266 (multiple myeloma) were obtained from American Type Culture Collection or as described previously [34] and cultured in R-10 medium. Cell lines were acquired between 2004 and 2010; HLA genotype and/or phenotype and expression of various cancer-associated antigens were confirmed by DNA sequencing, RT-PCR, and antibody staining, and lines displayed phenotypic characteristics of the tumor of origin. HEP2, HA2, and CM12 (normal human cells grown in culture) were purchased from ScienCell and NHEM10 cells from PromoCell.

Construction of J82-NY-ESO_157–165_ and SK-Mel-37-NY-ESO_157–165_ minigene-transfected cell lines has been described previously (as SLLMWITQC-minigene transfectants) [34].

J82-NY-ESO_157–165_ cells stably expressing enhanced green fluorescent protein (EGFP) (Clontech Laboratories, Inc., Mountain View, CA) were engineered using the pCGFP retroviral vector [37]. Production of infectious retroviral vector particles in Phoenix A cells and infection of cells were carried out as described [38]. This procedure was repeated twice, producing cells expressing high levels of GFP as determined by fluorescence microscopy (Leica Microsystems, GmbH, Wetzlar, Germany). The 5 % brightest cell population was isolated by a modified FACSAria II flow cytometer (BD BioSciences).

Peripheral blood lymphocytes (PBLs) were obtained from human healthy volunteers following Ficoll-Hypaque density gradient separation (Lymphoprep, Nycomed Pharma AS, Oslo, Norway).

Magnetic bead immunodepletion (Dynal) removed CD19+, CD4+, and CD14+ cells to obtain CD8+ ve T-cell populations.

### Quantitative RT-PCR on normal and neoplastic human cells

RNA was isolated from viably frozen specimens or cell lines growing in logarithmic phase using RNAqueous-4 PCR kits (Ambion corp, AM1914). cDNA was synthesized using iScript cDNA synthesis kits (Biorad Corp, 170-8891). qRT-PCR analysis was performed using standard Taqman—MGB technology and amplification conditions using an ABI 7500 FAST instrument (ABI-Life technologies), and the following ABI inventoried primer-probe sets: NY-ESO-1: HS00265824_m1; LAGE-1: HS00535628_m1; Gus-B: HS99999908_m1; β-Actin: HS99999903_m1. Amplifications were performed in triplicate; individual Ct values were determined (minimum of 2/3 replicates with % CV < 15 %) and average values reported. RQ (relative quantification) values for NY-ESO-1 and LAGE-1 transcripts were determined according to the formula RQ = 2^−ΔΔCt^, with ΔΔCt = ΔCt_sample_ − ΔCt_reference_, with ΔCt_sample_ = Ct_sample_ − Ct_sample_ normalizer and ΔCt_reference_ = Ct_reference_ − Ct_reference_ normalizer [39]. For all analyses, the melanoma cell line A375 (positive for NY-ESO-1 and LAGE-1) served as a reference; either Gus-B or β-Actin (for a subset of analyses) was used as the normalizer “housekeeping” gene.

### Engineering high-affinity TCRs and bi-specific ImmTACs

TCR isolation and engineering to produce ImmTAC reagents has been described previously [18, 40].

### Cytotoxicity and cytokine release assays

Cytotoxicity (LDH release) and IFNγ ELISpot assays were performed as previously described [18]. Multiple cytokine release (TNFα, IFNγ, and IL-2) was measured by Meso Scale Discovery (MSD) immunoassay following overnight incubation of CD8+ effector T cells and targets at a 1:1 ratio in the presence of 1 nM ImmTAC-NYE, in accordance with manufacturer’s instructions.

### IncuCyte and real-time quantification of cell killing

Target tumor cells were incubated with effector CD8+ T cells (5:1 *E*:*T*) in the presence or absence of ImmTAC-NYE. Images were taken every 10 min and the number of apoptotic cells per mm^2^ was quantified using the CellPlayer 96-well Kinetic Caspase 3/7 reagent and the IncuCyte-FLR-Platform (Essen Biosciences). The reagent is cleaved by activated Caspase 3/7 upon target cell apoptosis resulting in the release of the dye and green fluorescent staining of nuclear DNA. The cocktail of apoptotic drugs used as a positive control contains 10 μM stausporine, 2 μg/ml anisomycin, and 10 μM etoposide.

### TCR fluorescence labelling

TCR-NYE-wt, TCR-NYE-(29 nM), and TCR-NYE-(0.048 nM) were labelled with Alexa^®^ Fluor 680 carboxylic acid, succinimidyl ester as previously described [41]. Labelling was restricted to 2 fluors per molecule. Protein concentration and degree of labelling were determined by spectrophotometric analysis using a NanoDrop spectrophotometer (Thermo Scientific, Rockford, USA).

### Animal studies

Animal experiments were approved by The Norwegian Animal Research Authority and performed in accordance with the European Convention for the Protection of Vertebrates Used for Scientific Purposes. NOD-*scid* and NOD-*scid* IL2rγ*null* (NSG) mice (6–8 weeks old) were originally a gift of Prof. L. Shultz, Jackson Laboratories, Bar Harbour, USA, and were bred at the Gades Institute, University of Bergen. 5 × 10^6^ tumor cells were injected subcutaneously (s.c.) in a solution of PBS/Matrigel (1:1) bilaterally in flanks of mice (*n* = 2 per mouse). Tumor volumes were measured by digital calliper measurements using the formula: Volume = *π* (length × width × height)/6. Blood samples (100 μL) were acquired by submandibular bleeding.

### TCR imaging studies

When J82-NY-ESO_157–165_ tumors reached an average volume of 100–150 mm^3^, background fluorescence images of the mice (*n* = 4 per group) were acquired prior to intravenous (i.v.) injection with Alexa680 labelled TCRs (0.6 mg/kg) at 0 h and the mice imaged for TCR fluorescence at 4, 8, 24, 48, and 96 h.

### Tumor protection study in mice harboring human PBL xenografts

NSG mice were inoculated i.v. with 20 × 10^6^ human PBLs and randomized into study groups (equally distributed per percentage hu-CD45^+^CD3^+^). When mice demonstrated at least 10 % hu-CD3^+^ cells by flow cytometry (approx. week 3), SK-Mel-37-NY-ESO_157–165_ cells [34] were injected s.c. and mice treated i.v. with either ImmTAC-NYE (0.04 mg/kg, Q.D.) or PBS (equivalent volume) on days 1–5.

### ImmTAC efficacy studies in mice harboring cancer and human PBL xenografts

When J82-NY-ESO_157–165_^*GFP*^ tumors reached 50 mm^3^, 20 × 10^6^ human PBL per mouse were injected i.v. After mice demonstrated engraftment of human lymphocytes (at least 10 % hu-CD3^+^ cells) and tumor volumes reached 100–150 mm^3^, they were imaged for GFP fluorescence and randomized into study groups. Mice were then treated with ImmTAC-NYE, control ImmTAC-GAG (0.04 mg/kg, Q.D., i.v.), or PBS (equivalent volume, i.v.) for days 1–5 and tumors monitored weekly by time-domain optical imaging. The study was terminated 25 days following the first ImmTAC treatment.

### Optical imaging

Time-domain optical imaging was performed with Optix^®^ MX2 (ART Inc., Saint-Laurent, QC, Canada) as described previously [42]. Fluorescence images were acquired and analyzed with Optix^®^ Optiview™ (version 2.00; ART Inc.) and Optix^®^ Optiview™ (version 2.02.00, ART Inc.).

### Flow cytometric analysis of blood

Peripheral blood cells were analyzed by FACSCanto II (BD) using mouse anti-huCD3 (PE conjugate; clone SK7) and hu CD45 (FITC conjugate; clone 2D1) monoclonal antibodies (BD Immunocytometry). Human T-cell engraftment in peripheral blood was defined as the percentage of huCD45^+^CD3^+^ mononuclear cells (MNCs) in murine peripheral blood.

## Results

### Expression of NY-ESO-1 in normal and in neoplastic cells and tissues

We evaluated the expression of NY-ESO-1 and LAGE-1 in a panel of normal tissues and established cell lines, as well as a panel of primary tumor material from lung and ovarian cancer patients using quantitative real-time PCR. With the exception of low level LAGE expression in one normal tissue sample (SMC3), we did not detect transcripts for either NY-ESO-1 or LAGE-1 in 15 HLA-A2+ normal tissue cells, including melanocytes, hepatocytes, astrocytes, endothelial cells (aortic and dermal microvascular), fibroblasts (dermal and pulmonary), and epithelial cells (renal and ciliary) (Supplementary Table 1). Both NY-ESO-1 and LAGE-1 were broadly expressed at various levels and across a large number of tumor cell lines, including melanoma, myeloma, mesothelioma, lung, ovarian, and prostate cancer; among the tumor cell lines tested, only the colon cancer cell line Colo205 and the colorectal carcinoma HCT116 were negative for both antigens (Table 1 and Supplementary Table 2).

**Table 1:** Expression of NY-ESO-1 and LAGE-1 transcripts in primary NSCLC and ovarian cancer specimens, and in selected tumor cell lines

Tumor type	Sample ID	Gus-B	NY-ESO-1		LAGE-1	
		Avg. Ct	Avg. Ct	RQ	Avg. CT	RQ
Primary tumor samples						
Melanoma	A375	26.4	26.0	1	35.6	1
NSCLC	#25	24.1	ND	ND	ND	ND
NSCLC	#28	28.5	ND	ND	ND	ND
NSCLC	#29	26.5	27.0	0.54	28.7	127.3
NSCLC	#30	23.6	25.9	0.16	25.5	155
NSCLC	#31	24.9	ND	ND	ND	ND
NSCLC	#35	26.2	ND	ND	ND	ND
NSCLC	#79	28.3	ND	ND	ND	ND
NSCLC	#81	26.0	38.3	0.0002	32.6	6.27
NSCLC	#93	22.9	ND	ND	ND	ND
NSCLC	#95	24.4	ND	ND	ND	ND
Ovarian	#23	24.8	37.3	0.0001	28.5	46.2
Ovarian	#61	24.8	ND	ND	35.8	0.22
Ovarian	#65	25.8	39.0	0.0001	39.4	0.06
Ovarian	#79	20.1	35.2	0.014	35.2	0.017
Ovarian	#86	20.7	37.3	0.00001	38.5	0.0032
Ovarian	#90	22.9	37.5	0.00003	37.8	0.02
Ovarian	#91	23.4	ND	ND	38.3	0.024
Ovarian	#96	22.7	32.7	0.0008	24.7	159
Ovarian	#99	31.9	ND	ND	ND	ND
Ovarian	#103	24.3	ND	ND	ND	ND
Tumor cell lines						
Melanoma (reference)	A375	24.8	23.35	1	33	1
Melanoma	SK-Mel-37	23.8	38.4	0.00002	37.0	0.03
EBV transformed B cell from myeloma pt	IM9	23.3	25.2	0.10	23.9	195.3
Melanoma (reference)	A375	18.9*	22.1	1.000	31.2	1.000
Melanoma	Mel526	16.9*	37.2	0.00001	32.2	0.09
Multiple myeloma	U266	17.3*	20.2	1.2	21.2	311.8

*ND* not detected

* β-actin used as housekeeping gene

We further evaluated the expression of NY-ESO-1 and LAGE-1 in primary tumor specimens from ten NSCLC and ten ovarian cancer patients (Table 1). In NSCLC specimens, NY-ESO-1 and LAGE-1 transcripts were detected in 3 of 10 samples. In the ovarian cancer specimens, NY-ESO-1 was detected in 6 of 10 and LAGE-1 in 8 of 10 ovarian cancer specimens. NY-ESO-1 and LAGE-1 expression was not always concordant. Notably, as assessed by average Ct values, in some cases (NSCLC-#81, ovarian #23, #96), LAGE-1 mRNA was expressed at significantly higher levels than NY-ESO-1. Overall, 30 % of the NSCLC samples and 80 % of the ovarian specimens were positive for one or both antigens.

### Short-term redirected T-cell killing of NY-ESO-1 tumor-derived cell lines by ImmTAC-NYE

**Fig. 1:**
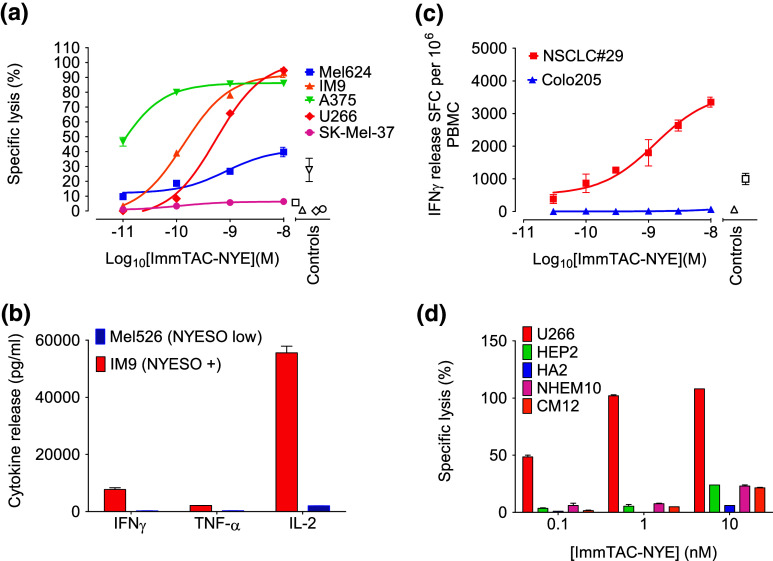
Activation and killing by ImmTAC-NYE redirected T cells against NY-ESO-1/LAGE-1 tumor-derived cell lines and freshly isolated tumor cells. **a** Redirected lysis of HLA-A2+, NY-ESO-1/LAGE+ tumor cell lines (as indicated in the figure key) by ImmTAC-NYE with purified CD8+ effector T cells. Controls: effectors and targets in the absence of ImmTAC-NYE, with the open symbol shapes matching the target cells indicated in the figure key. SK-Mel-37 cells present an average of 25 epitopes per cell, Mel624 cells an average of 45 and U266 cells an average of 5–10. **b** ImmTAC-NYE induces multiple cytokine release (TNFα, IFNγ, and IL-2) from CD8+ T cells in response to NY-ESO-positive IM9 cells; control cells are Mel526 (NY-ESO low). **c** IFNγ release from PBMCs in response to ImmTAC-NYE and freshly isolated LAGE-1+ lung cancer sample NSCLC#29 measured by ELISpot; Colo205 cells (antigen negative, HLA-A2^+^) are the specificity control. *Open symbols* represent targets and effectors without ImmTAC-NYE, with the symbol shapes matching the target cells indicated in the figure key. **d** ImmTAC-NYE does not lyse HLA-A2+, NY-ESO-1-/LAGE-1-negative primary human cells derived from normal tissues and expanded in culture. Purified CD8+ T cells were incubated with hepatocytes (HEP2), astrocytes (HA2), melanocytes (NHEM10), and cardiac myocytes (CM12) in the presence of 0.1, 1, and 10 nM ImmTAC-NYE. Antigen-positive U266 cells were included as a positive control. In **a** and **d,** lysis was determined by LDH release from target cells after 24 h, with an *E*:*T* of 10:1; *error bars* depict mean values ± SEM

ImmTAC-NYE has a binding affinity in the low pico-Molar range (approximately 50 pM) with a binding half-life of several hours; it has previously been shown to target two NY-ESO-1-/LAGE-1-positive tumor lines and activate T cells resulting in redirected killing [18]. The affinity-enhanced TCR is completely specific for the NY-ESO_157–165_ epitope presented by HLA-A*0201 demonstrating that specificity has not been lost during engineering (Supplementary Table 3). The ability of ImmTAC-NYE to redirect killing of additional HLA-A2*0201+, NY-ESO-1/LAGE-1+ tumor cell lines over 24 h was assessed by measuring LDH release from the target cells. Figure 1a shows a dose titration of ImmTAC-NYE in the presence of target tumor cells and CD8+ effector T cells. With the exception of the SK-Mel-37 cells, dose-dependent lysis of all the target cells was detected. No lysis was detected in the absence of ImmTAC-NYE. Figure 1b shows that ImmTAC-NYE induces the secretion of multiple cytokines (TNFα, IFNγ, and IL-2) from T cells in response to the IM9 cell line (NY-ESO-1/LAGE-1 high).

### ImmTAC-NYE activates T cells in the presence of freshly isolated tumor cells

To confirm that ImmTAC-NYE recognizes freshly isolated tumor cells in addition to tumor cell lines, we measured T-cell activation in response to lung cancer sample NSCLC#29, which expresses high levels of LAGE-1 (Table 1). Figure 1c shows that IFNγ is released from CD8 T cells in the presence of NSCLC#29 and ImmTAC-NYE, with a similar potency to redirected killing of tumor cell lines. Insufficient numbers of tumor cells were isolated to carry out a killing assay.

### ImmTAC-NYE is specific for its target peptide

The specificity of ImmTAC-NYE was investigated using HLA-A2*0201+, NY-ESO-1-/LAGE-1-negative primary human cells derived from normal tissues. The LDH release assay data shown in Fig. 1d demonstrate the lack of lysis of hepatocytes, cardiac myocytes, and melanocytes by concentrations of ImmTAC-NYE up to 1 nM; at 10 nM, a low level of lysis is observed. We have observed a slight loss of epitope specificity at high concentrations (10 nM and above) with other HLA-A2-specific ImmTACs, which is HLA-A2-dependent ([18] and unpublished data).

### Direct visualization of redirected tumor cell killing mediated by ImmTAC-NYE

**Fig. 2:**
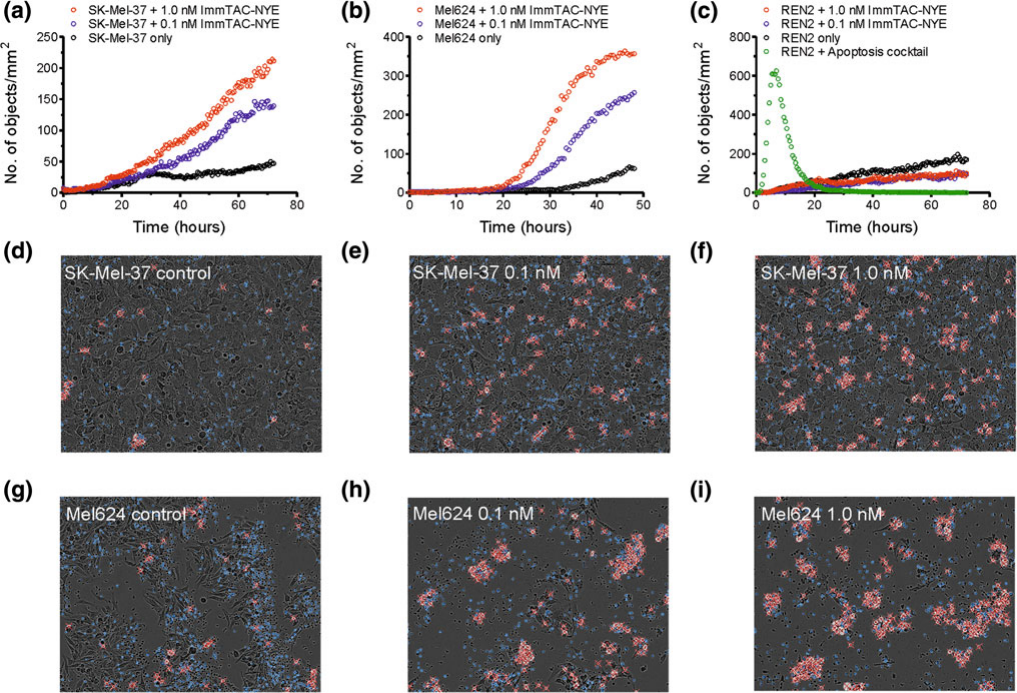
Target cell apoptosis induced by ImmTAC-NYE redirected T cells measured in real time. **a** SK-Mel-37, **b** Mel624 melanoma cells, or **c** REN2 normal renal epithelial cells were incubated with CD8+ cells in the presence or absence of ImmTAC-NYE and images taken at 10-min intervals. In **c** REN2, cells were also treated with a cocktail of drugs to induce apoptosis (apoptosis cocktail) to control for the presence of live cells. Representative end point images are shown for SK-Mel-37 (**d**–**f**, 72 h) and Mel624 (**g**, **h**, i 48 h) targets at the indicated concentrations of ImmTAC-NYE. *White flashes* marked with *red-crosses* represent cells undergoing apoptosis and are recorded as objects. Control: effectors and targets in the absence of ImmTAC-NYE

Killing of tumor cell lines over longer time periods was investigated using IncuCyte-FLR technology, enabling visualization of caspase 3/7-dependent apoptosis by microscopy at 37 °C in real time. Target tumor cells were incubated with effector CD8+ T cells in the presence or absence of ImmTAC-NYE and images taken at intervals of 10 min. The killing time course for SK-Mel-37 cells (HLA-A2*0201+, LAGE-1+), a cell line relatively resistant to lysis after 24 h (see Fig. 1a) is shown in Fig. 2a) in the presence of 0.1 nM and 1 nM ImmTAC-NYE. In agreement with the LDH assay (Fig. 1a), no significant killing was observed after 24 h. However, many apoptotic cells were present at 72 h, indicating that for some target cells (e.g., those that are resistant to lysis or with very low epitope levels), ImmTAC-NYE-induced killing requires in excess of 24 h. Figure 2b shows the killing time courses for Mel624 cells, again indicating that cell death occurs mainly after 24 h; the images taken at 48 h (Fig. 2h–i) confirm that close to 100 % of the cells have been killed at this time point. Figure 2c shows killing time courses for normal kidney epithelial cells (REN2) incubated with 0.1 and 1 nM ImmTAC-NYE over 72 h, confirming that normal cells are not killed in response to the ImmTAC even after 3 days. This is the first reported use of IncuCyte-FLR technology to measure CTL activity.

### In vivo imaging of tumors by soluble NY-ESO-specific TCRs

**Fig. 3:**
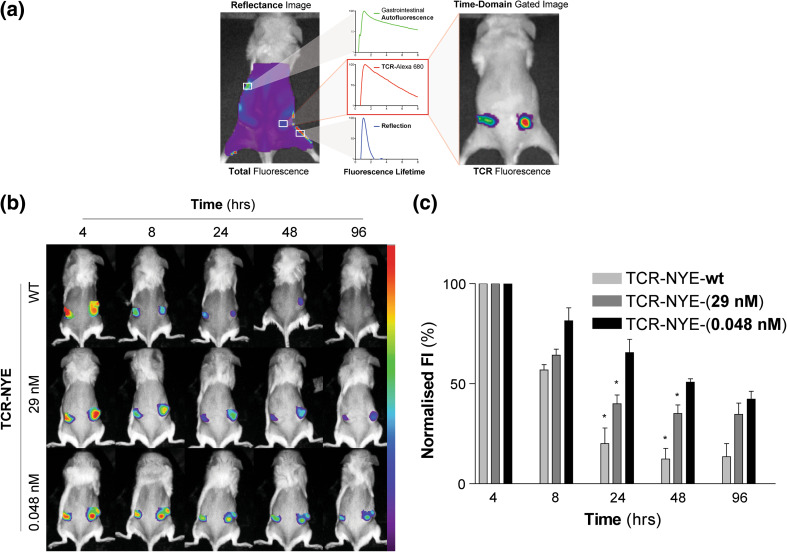
TCR-Alexa 680 targeting studies. **a** Mice xenografted with J82-NY-ESO_157–165_ cells were injected with Alexa 680 labelled TCR and analyzed at intervals using time-domain optical imaging. Images were then gated for the fluorescence lifetime of TCR-Alexa 680 and TCR-Alexa 680 photons quantified. **b** Representative gated fluorescence images of mice injected with TCR-NYE-wt, TCR-NYE-(29 nM), and TCR-NYE-(0.048 nM) over the time course. Fluorescence scale is the same for all mice. **c** Histogram of gated fluorescence normalized on 4-h results. *Error bars* present SEM. *p < 0.05

In vivo tumor targeting of NY-ESO-1 antigen was visualized using Alexa-fluor 680 labelled un-fused TCRs (rather than ImmTAC fusion proteins) in a xenograft model of J82-NY-ESO_157–165_, a human bladder cell line engineered to express the NY-ESO_157–165_ epitope [34]. Three TCRs were employed, all binding specifically to this epitope but differing in affinity: a) the wild-type TCR-NYE-wt, b) medium-affinity TCR-NYE-(29 nM), and c) high-affinity TCR-NYE-(0.048 nM); the affinities of the engineered TCRs are indicated in brackets, that is, 29 nM and 48 pM. Time-domain optical imaging of engrafted tumors was performed at intervals following administration of Alexa 680 labelled TCRs. The resulting images (Fig. 3a) were gated for fluorescence lifetimes of the TCR-Alexa 680 conjugates facilitating the distinction of specific mTCR-targeted fluorescence from background fluorescence. Mice inoculated with TCR-NYE-wt exhibited only transient tumoral fluorescence at the 4- and 8-h imaging time points before a return to background fluorescence levels at 48 h (Fig. 3b, c). Imaging of TCR-NYE-(29 nM)-treated mice demonstrated a similar pattern, although fluorescence intensities were higher (24 and 48 h) than those observed for the TCR-NYE-wt-treated mice. Only with mice injected with Alexa 680 labelled high-affinity TCR-NYE-(0.048 nM) were higher than background fluorescence levels observed throughout the time course; significantly, higher fluorescence intensities were noted in these mice at 24 and 48 h (*p* < 0.05), and even at 96 h, intensities were 2.5 times higher than background.

### ImmTAC-NYE therapy prevents the growth of NY-ESO epitope+ tumors in a xenografted mouse model

**Fig. 4:**
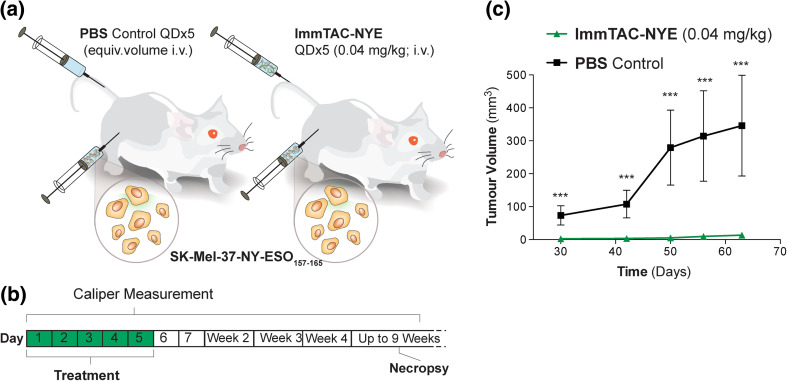
Tumor protection study in mice harboring human PBL xenografts. **a,** **b** Human PBL-engrafted mice were injected subcutaneously with SK-Mel-37-NY-ESO_157–165_ cells and treated intravenously with ImmTAC-NYE (*n* = 6) or PBS (*n* = 6) days 1–5. **c** Tumor volumes plotted against time. *Error bars* present SEM. ***p < 0.001

The evaluation of immuno-therapeutic agents in immunodeficient preclinical models typically requires subcutaneous (s.c.) co-injection of cancer and T cells or repeated injections [s.c. or intravenous (i.v.)] of high numbers of T cells in mice with pre-established tumors. However, these assays do not accurately reflect the potential of endogenous T cells to target cancer cells; for example, imaging studies over 4 days from the time of injection of T cells into tumor-bearing mice reported that less than 1 % of i.v. injected T cells home to tumors [42]. Recent studies have demonstrated the NSG mouse model to be sufficiently immunodeficient to permit the xenografting of functional immune systems [43]. Thus, we investigated whether this host would permit co-engraftment of both cancer cell lines and hu-PBLs, in order to develop a model in which the efficacy of ImmTAC-NYE can be evaluated in vivo. NSG mice were inoculated with hu-PBLs, and flow cytometry analyses showed the presence of robust populations of hu-CD45^+^CD3^+^ cells in xenografted recipients from week 3, with all mice exhibiting a hu-CD45^+^CD3^+^ cell population of at least 10 % of total peripheral blood lymphocytes. SK-Mel-37-NY-ESO_157–165_ cells were injected subcutaneously and treatment with either ImmTAC-NYE or PBS was initiated on the same day (Fig. 4a, b); a total of five doses of ImmTAC-NYE were administered.

While the tumors in PBS-treated mice underwent progressive growth with average volumes reaching 368 mm^3^ by day 50, palpable tumors were only just evident in the ImmTAC-NYE-treated mice at this time point (Fig. 4c; *p* < 0.001). Furthermore, once these tumors had been established, they developed with demonstrably slower growth kinetics only reaching average tumor volumes of 141 mm^3^ 3 months after treatment (data not shown).

### ImmTAC-NYE therapy targets established J82-NY-ESO_157–165_^*GFP*^ tumors in mice engrafted with human PBMC

**Fig. 5:**
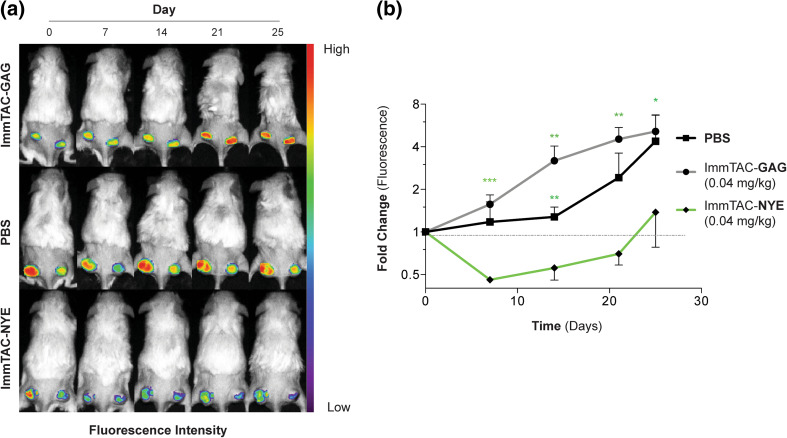
ImmTAC efficacy study in mice co-xenografted with human PBL and J82-NY-ESO_157–165_^*GFP*^ cells. **a** GFP gated fluorescence time course of representative mice xenografted with human PBL and J82-NY-ESO_157–165_^*GFP*^ cells and treated with ImmTAC-GAG (*n* = 4), ImmTAC-NYE (*n* = 4), or PBS (*n* = 4). **b** Average fold change in GFP-gated fluorescence for all animals over the time course. *Asterisks* indicate unpaired Student’s *t* test statistical analysis of the difference in GFP-gated fluorescence of ImmTAC-NYE- and ImmTAC-GAG-treated mice. *Error bars* present SEM. *p < 0.05, **p < 0.01, ***p < 0.001

Having demonstrated in vivo targeting of TCR-NYE to J82-NY-ESO_157–165_ cells and tumor prevention by ImmTAC-NYE in a xenograft model harboring both SK-Mel-37-NY-ESO_157–165_ cells and hu-CD45^+^CD3^+^ T-cell populations, we sought to evaluate anti-tumor efficacy of ImmTAC-NYE using optical imaging in pre-established tumors. J82-NY-ESO_157–165_^*GFP*^ cells were injected s.c. into the flanks of NSG mice, and when the tumors reached approximately 50 mm^3^, mice were inoculated with hu-PBL (Supplementary Figure 1). When recipient mice demonstrated at least 10 % T-cell chimerism (Supplementary Table 4) and tumor volumes of 100–150 mm^3^ (PBS, 124.9 ± 23.8 mm^3^; ImmTAC-GAG, 133.5 ± 25.8 mm^3^; ImmTAC-NYE 136.6 ± 4.7 mm^3^; one-way ANOVA *p* = 0.98), they were treated with ImmTAC-NYE (0.04 mg/kg), control ImmTAC-GAG (specific for an HLA-A2 presented HIV epitope) (0.04 mg/kg) or PBS for 5 days, and tumor viability monitored by fluorescence imaging (Supplementary Figure 2). Treatment of control groups with either ImmTAC-GAG or PBS did not affect the tumor growth of the J82-NY-ESO_157–165_^*GFP*^ cells as demonstrated by GFP fluorescence intensity (Fig. 5a, b). In contrast, mice treated with ImmTAC-NYE exhibited significantly reduced GFP fluorescence on Day 7 when compared with pre-treatment Day 0 fluorescence and ImmTAC-GAG or PBS controls on Day 7 (*p* < 0.001). By days 14 and 21, the GFP fluorescence intensity values of J82-NY-ESO_157–165_^*GFP*^ tumors in ImmTAC-NYE-treated mice increased from day 7 nadirs but remained significantly lower than either of the control groups (*p* < 0.01).

## Discussion

The current study was performed to investigate the utility of a bi-specific ImmTAC reagent targeting an epitope common to both NY-ESO-1 and LAGE-1 for cancer immunotherapy. The expression patterns of both NY-ESO-1 and LAGE-1 in tumors identify these proteins as suitable targets for the treatment of a wide variety of malignancies. NY-ESO-1 and LAGE-1 are members of the cancer testis class of tumor-associated antigens, which has the “cleanest” expression profile in normal tissue. Since these antigens are intracellular, they are not accessible to conventional therapeutic antibodies and targeting of MHC-presented peptide epitopes provides an opportunity for tumor-directed immunotherapy [35, 44].

RT-PCR studies have detected NY-ESO-1 or LAGE-1 transcripts in the testis, placenta, and ovary; other normal human tissues completely lacked expression, with the exception of one qRT-PCR study which also identified low levels of NY-ESO-1 mRNA in liver and pancreas [20, 22, 45]. In broad agreement with previous data, qRT-PCR analyses performed in the current study did not detect NY-ESO-1 or LAGE-1 transcripts in several normal human tissues including hepatocytes, melanocytes, and a range of fibroblasts and endothelial cells derived from different organs. In contrast, NY-ESO-1 and/or LAGE-1 transcript expression was detected not only in cell lines derived from a variety of tumor types but also in primary tumors of ovarian cancer and NSCLC. In agreement with other studies [25, 35, 46, 47], there was general overlap in NY-ESO-1 and LAGE-1 mRNA expression, although a subset of samples was identified which expressed only one of the antigens (Table 1). Particularly for the ovarian cancer specimens, LAGE-1 expression was detected at higher RNA levels and at a greater frequency than NY-ESO-1, suggesting that LAGE-1 may be a superior target antigen for cancer immunotherapy in at least some tumor types. Andrade et al. [24] made a similar observation in multiple myeloma (49 % LAGE-1 vs. 33 % NY-ESO). A previous study detected presentation of the NY-ESO-1_157–165_ epitope by the SK-Mel-37 and Mel 624 cell lines [34], which express far higher levels of RNA for LAGE-1 than for NY-ESO-1 (Table 1), confirming that this peptide can be processed efficiently from LAGE-1.

We have engineered three soluble mTCRs specific for the NY-ESO-1_157–165_ epitope, TCR-NYE-wt (the wild-type affinity TCR), and two affinity matured variants TCR-NYE-(29 nM) and TCR-NYE-(0.048 nM). Since previous studies have shown the importance of mTCR affinity for ImmTAC potency [18], the highest affinity ImmTAC-NYE was evaluated in vitro and in vivo. ImmTAC-NYE was able to activate and redirect normal CD8+ T cells to a range of cell lines derived from melanoma (A375, Mel624, and SK-Mel-37), myeloma (U266), and EBV transformed B cells (IM9), in addition to freshly isolated lung cancer cells. The demonstration that the redirected cell lysis was limited to those cells expressing endogenous NY-ESO-1 and/or LAGE-1 proteins [34] was essential to exclude the possibility of non-specific toxicity against normal tissues in a clinical setting. While significant lysis of the majority of the tumor cell lines occurred during the first 24 h, the IncuCyte real-time imaging system demonstrated that some cell lines required longer periods for caspase 3/7-dependent apoptosis to occur. One possible explanation for the observed variation in cell lysis is the difference in density of target epitopes on the surface of each cell line; previous studies using TCR-NYE-(0.048 nM) reported the presence of 25 and 45 epitopes/cell on the SK-Mel-37 and Mel624 cell lines respectively [34], and U266 cells have 5–10 epitopes per cell (unpublished data). Other factors which may influence the rate of cell killing are firstly susceptibility of individual tumor cell lines to lysis and secondly the presence of cofactors such as CD80 and CD86 on the tumor cells which may augment killing.

An essential element for the efficacy of targeted TCR therapy is the effective delivery of engineered reagents to the tumor site. Such targeting of biologics has previously been demonstrated in vivo employing molecular imaging in both clinical and preclinical settings [48]. In particular, fluorescence reflectance molecular imaging has typically been employed preclinically owing to its high sensitivity [49], low cost, and potential for rapid in vivo screening. A major challenge for this strategy is to achieve sufficient target-to-background ratios to evaluate specific targeting over endogenous background auto-fluorescence and non-specific binding. However, the density of specific MHC/peptide complexes on the cell surface is usually low (between 10 and 150 epitopes per cell) ([34] and unpublished data), thwarting the use of TCRs in conventional fluorescence imaging.

In contrast to conventional imaging, fluorescence lifetime or time-domain imaging can discriminate between fluorophores with different lifetime decay rates [50]. Time-domain optical imaging has been employed with great success in distinguishing fluorescently labelled monoclonal antibodies bound to target antigens from both auto-fluorescence [41] and background fluorescence from unbound antibodies [51]. In the current study, the administration of three near-infrared-labelled TCR-NYEs of varying affinities, combined with the use of time-domain optical imaging techniques, confirmed targeting of the mTCRs to tumors presenting the NY-ESO-1_157–165_ epitope.

To evaluate in vivo efficacy of ImmTAC-NYE, we utilized NSG mice engrafted with human PBL in both engraftment and established tumor models. We demonstrated that a short course (5-day administration) of ImmTAC-NYE substantially delayed engraftment of SK-Mel-37-NY-ESO_157–165_ tumor cells, with tumors just detectable in treated mice 50 days post-implantation, while tumors had reached an average volume of 368 mm^3^ in the control treated mice. In an established tumor model using J82 tumor cells engineered to express NY-ESO_157–165_ and GFP, we observed that five daily doses of ImmTAC-NYE resulted in a significant reduction in GFP fluorescence 7 days post-initiation of therapy compared to control treated mice, indicating a substantial reduction in tumor cell viability. An issue with this particular cell line in vivo was its inherent slow growth kinetics, which made differentiation of therapeutic effect in this pilot study impossible by calliper measurements. The significant reduction in fluorescence demonstrated with only five doses of ImmTAC-NYE by time-domain optical imaging endorses both application of this model and fluorescence lifetime gating in further efficacy strategies.

In xenografted animal models which have investigated ImmTAC efficacy (this study and [18]), inhibition of tumor growth continues for long periods of time (over 5 weeks) after the final dose of reagent. ImmTAC-activated T cells release soluble factors including interferon-γ, IL-2 and TNFα (Fig. 1b), which can not only attract additional T cells and other effector immune cells to the tumor site but also promote components of the death receptor pathway in the tumor cells [52], which can result in long-term anti-tumor activity. In cancer patients, it is probable that mechanisms such as epitope spreading (activation of endogenous T cells specific for other tumor epitopes) will occur, following cross-presentation of antigens from lysed tumor cells by dendritic cells. Indeed, we have demonstrated cross-presentation induced by ImmTAC redirected tumor cell killing in vitro (manuscript in preparation). These mechanisms have the potential to stimulate a prolonged anti-tumor immune response capable of breaking tumor tolerance. In addition, we have previously demonstrated serial killing of several tumor cells by a single ImmTAC-activated T cell, which may partly account for the high potency of these reagents [18]. It is not known whether the PBMC-engrafted mice have functioning dendritic cells and therefore whether cross-presentation and epitope spreading can occur in this model system.

ImmTACs have been shown to detect low densities of cell surface epitope (as few as 10 per cell), in contrast to naturally occurring tumor-specific T cells which require higher levels of target epitope to become activated [18]. Thus, ImmTACs have the potential to kill tumors which are not recognized by low-avidity T cells in patients, for example responses induced by cancer vaccines, since higher avidity CTL specific for self-peptides have been removed by negative thymic selection or inactivated by peripheral tolerance mechanisms. The ability of ImmTACs to detect a low density of pMHCs and to activate immune cells independently of co-receptors and other regulatory cells could circumvent immune tolerance in the tumor microenvironment and overcome inhibitory mechanisms such as MHC down-regulation or the presence of Tregs [8, 9]. Decreased levels of MHC (relative to normal tissue cells) are frequently observed in tumors, whereas complete loss of both chromosomal loci encoding MHC and associated processing machinery is a relatively rare event (unpublished observations and [53, 54]). Only patients with tumors positive for both NY-ESO-1/LAGE-1 and HLA-A*0201 are eligible for treatment with ImmTAC-NYE; we are currently isolating NY-ESO-specific TCRs with different HLA restrictions, in order to expand the target patient population. To conclude, we have demonstrated specific and potent ImmTAC-NYE redirected killing of NY-ESO-1 and LAGE-1 tumor cells both in vitro and in vivo, supporting the clinical development of this bi-specific reagent.

### Electronic supplementary material

Below is the link to the electronic supplementary material.

Supplementary material 1 (PDF 601 kb)
