# Supplementary material for: Bi-specific TCR-anti CD3 redirected T-cell targeting of NY-ESO-1- and LAGE-1-positive tumors
Source: Cancer Immunol Immunother. 2012 Dec 22;62(4):773–85. doi: 10.1007/s00262-012-1384-4 (PMC3624013; doi:10.1007/s00262-012-1384-4)
Supplement: Supplementary file 1 — Supplementary material 1 (PDF 601 kb) [file 262_2012_1384_MOESM1_ESM.pdf]

Supplementary Data

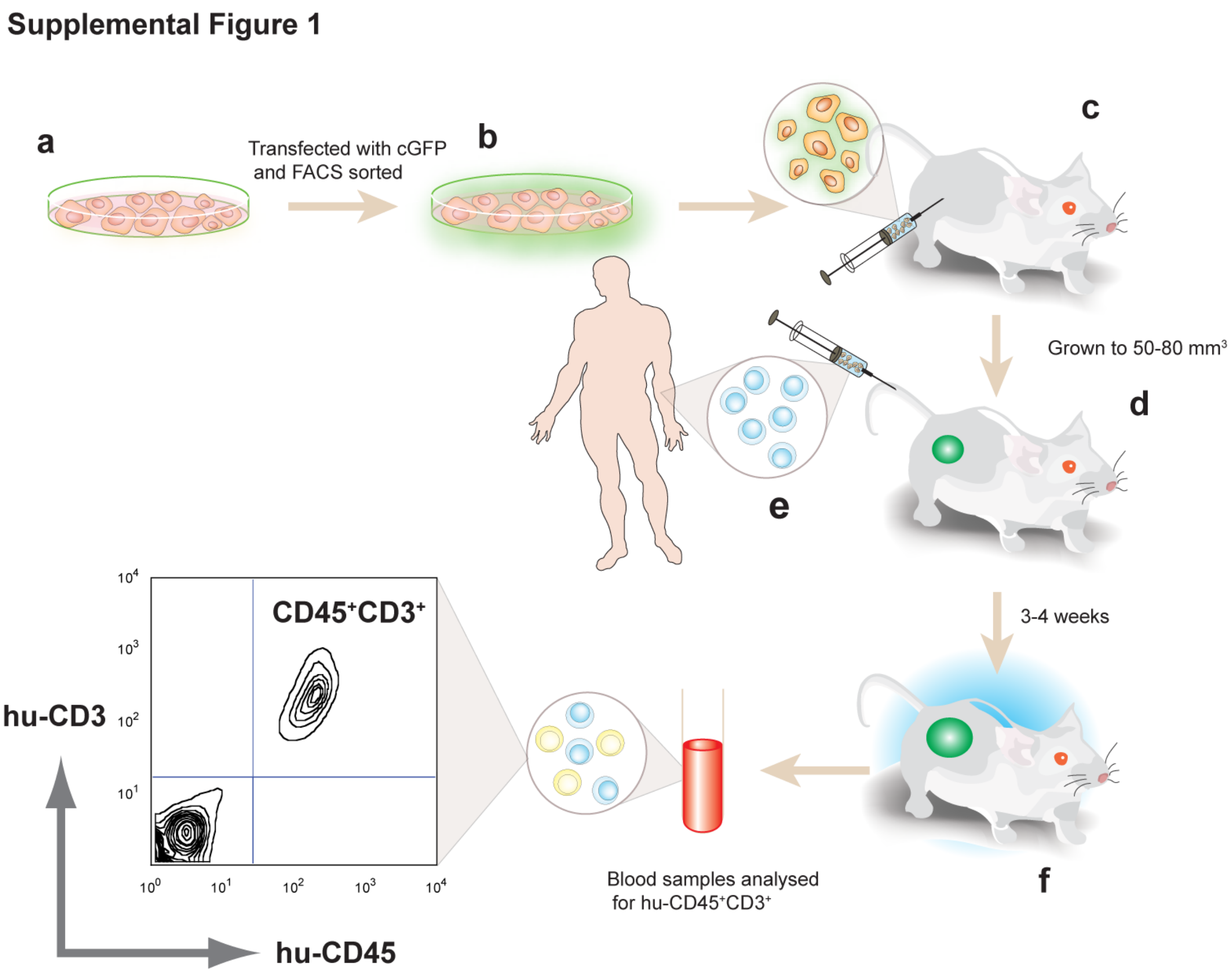

Supplemental Figure 1: ImmTAC efficacy study design. (a) J82-NY-ESO$_{157\text{-}165}$ cells are stably transfected with cGFP expression. (b) Cells are then sorted for high expression of GFP. (c) Mice are injected subcutaneously with 5×10^6 J82-NY-ESO$_{157\text{-}165}$ cells and (d) grown to approximately 50-80 mm^3. (e) Mice are administered intravenously with 20×10^6 PBLs isolated from a healthy donor and permitted to engraft over 3-4 weeks after which time (f) blood samples are analysed by flow cytometry for the presence of human CD45$^+$CD3$^+$ cells.

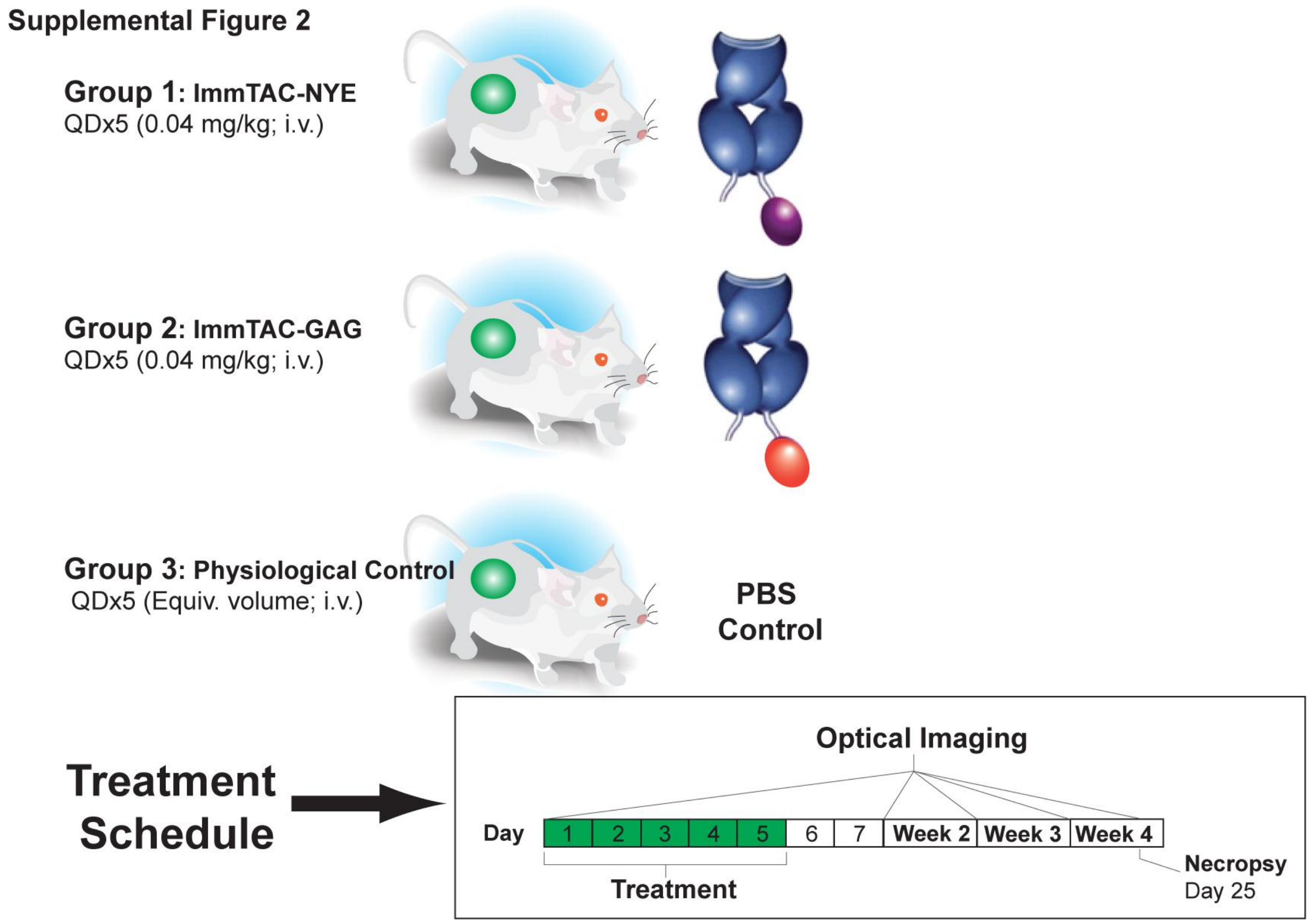

Supplemental Figure 2: ImmTAC efficacy study treatment and imaging schedule. Mice (*n* = 12) xenografted bilaterally with J82-NY-ESO$_{157\text{-}165}^{GFP}$ and human PBL were randomised by tumour volume, % human CD45^{+}CD3^{+} cells and GFP fluorescence and treated with ImmTAC-NYE (*n* = 4), ImmTAC-GAG (*n* = 4), or PBS control on Day 1-5 as indicated. Imaging was performed prior to imaging and on days 7, 14, 21 and at termination of the study on day 25.

Supplemental Table 1

Cells	Cell Type	Transcript: Reference				
		Reference	NY-ESO-1		LAGE-1	
		Avg. Ct.	Avg. Ct	RQ	Avg. CT	RQ
A375	Melanoma (Reference sample)	24.8*	23.35	1.0	33.01	1.0
HNPCEpIC (CIL1)	Ciliary epithelial cells	25.1*	ND	ND	ND	ND
HDMEC2	Dermal microvascular endothelial cells	23.9*	ND	ND	ND	ND
HDMEC3	Dermal microvascular endothelial cells	16.4**	ND	ND	ND	ND
HDF1	Dermal fibroblasts	23.7*	ND	ND	ND	ND
HPF1	Pulmonary fibroblasts	24.7*	ND	ND	ND	ND
HRPEpiC (HRP1)	Retinal pigmented epithelial cells	24.2*	ND	ND	ND	ND
REpiC (REN2)	Renal epithelial cells	24.5*	ND	ND	ND	ND
HA3	Astrocytes	25*	ND	ND	ND	ND
HA2	Astrocytes	19.4**	ND	ND	ND	ND
HAoEC1	Aortic endothelial cells	24.9*	ND	ND	ND	ND
HNEM N10	Epidermal melanocyte	34.9**	ND	ND	ND	ND
HNEM N5	Epidermal melanocyte	17.2**	ND	ND	ND	ND
HNEM N3	Epidermal melanocyte	17.9**	ND	ND	ND	ND
HEP2	Human Hepatocytes	15.5**	ND	ND	ND	ND
SMC3	Normal human smooth muscle cells	15.3**	ND	ND	36.8	0.002

Supplemental table 1: Expression of NY-ESO-1 and LAGE-1 in primary normal tissues: *Gus-B; **b-actin.
ND: Not detected

Supplemental Table 2

Cell line	Cell Type	Transcript: Reference Avg. Ct.	NY-ESO-1 Avg. Ct	NY-ESO-1 RQ	LAGE-1 Avg. CT	LAGE-1 RQ
A375	Melanoma (Reference sample)	18.9**	22.1	1.000	31.2	1.000
Mel 38	Melanoma	19.5**	21.2	2.9	24.5	150.3
H929	Multiple myeloma	17.8**	25.0	0.08	29.1	1.51
EJM	multiple myeloma	23.7*	22.2	1.1	22.8	541.1
H1975	Non-small cell lung cancer	15.0**	35.5	0.00001	37.4	0.001
H226	Lung squamous carcinoma	15.9**	37.7	0.000003	ND	ND
OV79	Ovarian carcinoma	15.1**	35.9	0.00001	34.4	0.007
NALM-6	Pre- B cell leukemia	17.3**	36.7	0.00002	35.4	0.02
M108	Mesothelioma	16.6**	ND	ND	37.2	0.003
LnCAP	Prostate cancer	20**	36.7	0.00010	34.5	0.147
PC3	Prostate cancer	16.6**	ND	ND	ND	ND
PP	EBV transformed B cell line	14.7**	ND	ND	ND	ND
VERD	EBV transformed B cell line	16.9**	ND	ND	ND	ND
Colo205	Colon carcimoma	20.8**	ND	ND	ND	ND
HCT116	Colorectal carcinoma	21.4**	ND	ND	ND	ND

Supplemental table 2: Expression of NY-ESO-1 and LAGE-1 in established tumor cell lines. *Gus-B; **b actin.
ND: Not detected

Supplemental Table 3

Epitope	Antigen	KD
HLA-A2 - SLLMWITQC	NY-ESO	48 pM
HLA-A2 - KIFGSLAFL	Her-2/Neu	no binding*
HLA-A2 - ALGSTAPPV	MUC-1	no binding*
HLA-A2 - LLGDLFGV	hMDM-2	no binding*
HLA-A2 - YLQVNSLQTV	Telomerase (988-997)	no binding*
HLA-A2 - LTLGEFLKL	Proteinase PRI	no binding*
HLA-A2 - ILLRDAGLV	TRAG-3	no binding*
HLA-A2 - ALQPGTALL	PSCA	no binding*
HLA-A2 - LLHETDSAV	PSMA	no binding*
HLA-A2 - YMDGTMSQV	Tyrosinase	no binding*
HLA-A2 - ELAGIGILTV	Melan-A	no binding*
HLA-A2 - YLSGANLNL	CEA	no binding*
HLA-A2 - NLVPMVATV	CMV	no binding*
HLA-A2 - RMFPNAPYL	WT-1 (126-134)	no binding*
HLA-A2 - ILAKFLHWL	Telomerase (540-548)	no binding*

Supplemental table 3: Binding of TCR-NYE-(0.048 nM) to purified HLA-A2-peptides measured by Surface Plasmon Resonance (SPR).
* Limit of detection for SPR binding is approximately K_D500μM.

Supplemental Table 4

	**Group** (% human $CD45^{+}CD3^{+}$)		
**Mouse no.**	PBS	ImmTAC-GAG	ImmTAC-NYE
1	12.4	22.3	19.8
2	16.2	21.4	16.1
3	24.7	14.7	12.9
4	18.1	10.2	20.1
Mean ± SD	17.9 ± 5.1	17.1 ± 5.7	17.2 ± 3.4
One-way ANOVA p = 0.975			

Supplemental table 4: Percentage human $CD45^{+}CD3^{+}$chimerism in J82-NY-$ESO_{157\text{-}165}^{GFP}$ at study initiation.
